# Exercise-Induced Hypoxemia in Endurance Athletes: Consequences for Altitude Exposure

**DOI:** 10.3389/fspor.2021.663674

**Published:** 2021-04-26

**Authors:** Fabienne Durand, Antoine Raberin

**Affiliations:** ^1^Images Espace Dev, Université de Perpignan Via Domitia, Perpignan, France; ^2^Laboratoire IMS, Université de Bordeaux, Bordeaux, France

**Keywords:** endurance, performance, exercise induced hypoxemia, altitude, O_2_ desaturation, altitude/hypoxia training, endurance performance

## Abstract

Exercise-induced hypoxemia (EIH) is well-described in endurance-trained athletes during both maximal and submaximal exercise intensities. Despite the drop in oxygen (O_2_) saturation and provided that training volumes are similar, athletes who experience EIH nevertheless produce the same endurance performance in normoxia as athletes without EIH. This lack of a difference prompted trainers to consider that the phenomenon was not relevant to performance but also suggested that a specific adaptation to exercise is present in EIH athletes. Even though the causes of EIH have been extensively studied, its consequences have not been fully characterized. With the development of endurance outdoor activities and altitude/hypoxia training, athletes often train and/or compete in this stressful environment with a decrease in the partial pressure of inspired O_2_ (due to the drop in barometric pressure). Thus, one can reasonably hypothesize that EIH athletes can specifically adapt to hypoxemic episodes during exercise at altitude. Although our knowledge of the interactions between EIH and acute exposure to hypoxia has improved over the last 10 years, many questions have yet to be addressed. Firstly, endurance performance during acute exposure to altitude appears to be more impaired in EIH vs. non-EIH athletes but the corresponding physiological mechanisms are not fully understood. Secondly, we lack information on the consequences of EIH during chronic exposure to altitude. Here, we (i) review research on the consequences of EIH under acute hypoxic conditions, (ii) highlight unresolved questions about EIH and chronic hypoxic exposure, and (iii) suggest perspectives for improving endurance training.

## Introduction

For many years, cardiovascular responses and muscle metabolism were the only acknowledged limiting factors in exercise performance. However, research has now clearly shown that the respiratory system is far from perfect and does not always have the capacity to meet the demands of intense endurance training (Amann, 2012). Dempsey et al.'s (2020) elegant review analyses the consequences of an “underpowered” respiratory system in endurance-trained athletes, where the excessive strain placed on to the respiratory system (relative to its capacity) leads to several problems, among them a phenomenon called exercise-induced hypoxemia (EIH).

EIH was first described in detail by Dempsey et al. in the 1980s. EIH occurs in some (but not all) endurance-trained athletes performing exercise at sea level (i.e. under normoxic conditions) (Dempsey et al., 1984; Dempsey and Wagner, 1999). The phenomenon involves a decrease in arterial oxygen pressure (PaO_2_) and a concomitant drop in arterial oxygen saturation (SaO_2_) between rest and maximal exercise. This hypoxemia is associated with abnormal gas exchange, as reflected by an increase in the alveolo-arterial oxygen pressure difference [D(A-a)O_2_] (Dempsey and Wagner, 1999; Prefaut et al., 2000). Since PaO_2_ and SaO_2_, together with the hemoglobin concentration, impact the arterial O_2_ content (CaO_2_), the oxygen (O_2_) supply to the muscles and thus aerobic exercise performance at sea level are impacted. In this context, EIH has attracted interest from sports scientists and physiologists.

## EIH Definition and Physiological Determinants

Historically, EIH has been studied with invasive measurements of PaO_2_ and SaO_2_. The O_2_ dissociation curve for hemoglobin shows that the fall in PaO_2_ is linked to O_2_ desaturation, i.e., a fall in SaO_2_. Many studies have found that SaO_2_ ranges from 87 to 94% in EIH athletes (Powers et al., 1984, 1988; Sheel et al., 2001; Stewart et al., 2003). This involved that the fall of PaO_2_ is large enough to be located to the sloping part of the Barcroft's curve. Several physiological aspects must be considered in the strict definition of EIH. A fall in PaO_2_ of at least 10 mmHg between rest and maximal exercise is required, considering that (i) hypoxemia at rest is defined as a fall of 5 mmHg (Préfaut et al., 1988), and (ii) there is a 2–3 mmHg error in the PaO_2_ measurement during a maximal exercise test (due to the shift in Barcroft's curve with increasing temperature) (Holmgren and McIlroy, 1964). However, PaO_2_ is not the major determinant of CaO_2_ as opposed to SaO_2_, and due to the flat part of the Barcroft's curve some drop in PaO_2_ could not induced any fall in SaO_2_. So, SaO_2_ may be a greater marker to define EIH and the first studies to use SaO_2_ as a tool for measuring EIH considered SaO_2_ < 94% to be abnormal and so set thresholds with a certain margin of error (Powers et al., 1988; Harms and Stager, 1995).

After showing that VO_2_max decreases when SaO_2_ goes below 95%, Dempsey and Wagner (1999) suggested the following classification: mild EIH for SaO_2_ values between 95 and 93%, moderate EIH for 93–88%, and severe EIH for below 88%. To avoid invasive method, the strong relationship between SaO_2_ and oxygen pulse saturation values (SpO_2_) at rest and during exercise was considered. The use of pulse oximetry to study EIH was validated (Mollard et al., 2010). Considering inter-individual differences in the resting SpO_2_ values, taking into account (i) the inherent rightward shift of Barcroft's curve during exercise, and (ii) the accuracy of oximeters, Prefaut et al. (2000) defined EIH as a drop in SpO_2_ of at least 4% between rest and exercise. Since transient SpO_2_ drops are typically observed during exercise (Préfaut et al., 1994), the persistent nature of the desaturation must also be taken into account. Hence, the most suitable definition of EIH is a drop of at least 4% in SpO_2_ during one or more of the last three steps in an incremental exercise test to exhaustion (Durand et al., 2000; Prefaut et al., 2000). However, steps during a test to exhaustion vary in duration and workload charge, thus to avoid confusion between transient SpO_2_ drops and EIH a minimum of 3 min of desaturation could be recommend. The magnitude of the fall in SpO_2_ reported during EIH is the same as for SaO_2_ (Grataloup et al., 2005; Stewart and Pickering, 2007; Kyparos et al., 2012; Riganas et al., 2019).

Since the 1990s, several studies showed that athletes with EIH (EIH athletes) and without (NEIH athletes) with similar training volumes have the same VO_2_max (Legrand et al., 2005; Gaston et al., 2016; Raberin et al., 2019a; Durand et al., 2020). This prompted leading sports scientists to dismiss EIH as not being relevant. However, beyond a SaO_2_ decrement of 4% in men and 3% in women, each additional 1% decrement results in a 1% decrement in VO_2_max (Powers et al., 1993; Harms et al., 2000). EIH appears to be a multifactorial phenomenon, with the involvement of ventilation/perfusion mismatch, shunts (intrapulmonary or intracardiac), relative hypoventilation and diffusion limitation (Dempsey and Wagner, 1999). The contribution of each factor is still unclear, although it could partly depend on the intensity of the exercise (Durand et al., 2000). Anyway the purpose of this review is not to discuss around the pathophysiology of EIH but its consequences.

## Prevalence of EIH at Sea-Level

Few researchers have focused on the prevalence of EIH and an early study reported that 52% of male athletes with a VO_2_max > 68 ml^.^min^−1.^kg^−1^ displayed EIH during an incremental exercise test on a cycle ergometer (Powers et al., 1988). Some publication have reported a similar prevalence (Mucci et al., 2000; Connes et al., 2004a; Grataloup et al., 2005; Gaston et al., 2016), whereas others found higher values (60–75%) (Connes et al., 2004b; Guenette et al., 2004; Connes and Boucher, 2010) or lower values (30–35%) (Sheel et al., 2000; Alis et al., 2015). Recently, a prevalence of 70% was reported in a large cohort (*n* = 79) of male athletes with a VO_2_max > 68 ml.min^−1^.kg^−1^ performing a progressive-grade treadmill exercise test to exhaustion (Constantini et al., 2017). Due to a dysanapsis phenomenon with smaller relative lung size, women would be more prone to develop EIH than men (St Croix et al., 1998; Dominelli et al., 2013). Richards et al. (2004) reported that 67% of young active women (VO_2_max range: 28–61 ml^.^kg^−1.^min^−1^) present EIH during a cycle test to exhaustion.

EIH is also present at submaximal exercise intensities in some individuals (Dominelli and Sheel, 2018). In this context the “Demand vs. Capacity” theory is unable to explain the phenomenon. There is some evidence to show that hypoxemia during submaximal exercise is the result of relative alveolar hypoventilation while a non-ventilatory mechanism seemed to affect gas exchanges beyond the second ventilatory threshold and thereby enhancing EIH (Durand et al., 2000). The relative hypoventilation is probably at least a result of metabolic changes and different ventilatory responsiveness due to a high level of endurance training (Brooks and Mercier, 1994; Mucci et al., 1998; Granger et al., 2020).

Despite the presence of this large body of evidence, one group of researchers has claimed that EIH is a fallacy because most studies did not correct hypoxemia considering the rightward shift of the oxy-hemoglobin dissociation curve due to temperature, CO_2_ and pH (Scroop and Shipp, 2010). Furthermore, EIH is typically observed during exercises in a laboratory at sea level. On this basis, studies have suggested that EIH reduces performance and induces fatigue (Powers et al., 1988; Romer et al., 2006). However, laboratory conditions do not reflect ecological condition of training or competition, and so EIH and its consequences have not been studied during real training sessions. As a result, the athletes and coaches have likely underestimated the importance of EIH.

## EIH in Altitude

Some endurance athletes are required to compete at altitude. For example, trail running has become particularly popular in recent years and the craze is prompting more and more athletes to exercise at moderate altitude, often without acclimatization. In this context of acute exposure to moderate altitude, it has been reported that EIH athletes perform less well than NEIH athletes (Chapman et al., 1999; Gaston et al., 2016; Raberin et al., 2019a). Studies that monitored EIH during an acute exposure to hypoxia were reviewed in Table 1. These studies must have to include a control group in order to investigate the interaction between EIH and exposure to altitude during acute exposure. The studies were carried out under simulated altitudes of 1000–3000 m (Chapman et al., 1999; Grataloup et al., 2007; Raberin et al., 2019a), or under naturally hypoxic conditions at 2150 m (Gaston et al., 2016). All the studies found that the drop in VO_2_max during exercise at altitude was greater in EIH athletes. According to some researchers, this drop could be explained (at least in part) by the fact that the EIH athletes had a lower maximal heart rate (HR_max_) at altitudes of 2150 m (Gaston et al., 2016), 2400 m (Raberin et al., 2019a), 3000 m (Grataloup et al., 2007), and 5000 m (Benoit et al., 2003). In contrast, Chapman et al. (1999) did not find a difference in HR_max_ between EIH and NEIH athletes at an altitude of 1000 m. Some researchers have suggested that the reduction in VO_2_max in EIH athletes at altitude is associated with the fall in SaO_2_ observed at sea level (Gavin et al., 1998; Chapman et al., 1999, 2011). Indeed, SpO_2_ during exercise at altitude is lower in EIH athletes than in NEIH athletes in some studies (Grataloup et al., 2007; Raberin et al., 2019a). A decrease in the ventilatory response to hypoxia can likely explain the lower SaO_2_ measured in EIH athletes. EIH athletes might fail to have an adequate ventilation during exercise at altitude, which might contribute to hypoxemia (Harms and Stager, 1995; Derchak et al., 2000). However, some studies did not find a relationship between the ventilatory response to hypoxia and SaO_2_ (Hopkins and McKenzie, 1989; Guenette et al., 2004). Recent studies of the ventilatory response to hypoxia and hypercapnia in EIH athletes have generated interesting results (Granger et al., 2020). Indeed, athletes with the lowest SpO_2_ during exercise showed a lower ventilatory response to hypercapnia (but not to hypoxia) than athletes with a higher SpO_2_. This blunted hypercapnic ventilatory response might have consequences on ventilatory acclimatization to altitude. Nevertheless, other studies did not find greater hypoxemia values in EIH athletes (vs. NEIH athletes) during hypoxic exercise (Benoit et al., 2003; Verges et al., 2005; Gaston et al., 2016). In the study by Gaston et al. (2016), the larger decrease in VO_2_max in EIH athletes (22 vs. 16% in NEIH athletes) could not be wholly explained by a greater reduction in HR_max_ and so suggested the involvement of another factor and probably specific adaptation to hypoxia in EIH athletes. More recently, it was reported that EIH athletes displayed exacerbated changes in cerebral deoxygenation and a low limb muscle blood volume at sea level; these results were accentuated by the severity of O_2_ arterial desaturation, and in contrast to NEIH athletes, were the same in hypoxia and normoxia (Raberin et al., 2019a).

**Table 1:** Summary findings of studies of EIH athletes exercising under acute hypoxic conditions.

**Study**	**Participants**	**Protocol**	**Exercise test**	**Results**
Benoit et al. (2003)	Five EIH and seven non-EIH athletes (runners or cyclists)	Two maximal ramp tests in normoxia and hypoxia (FiO_2_ = 10.4%)	A cycling maximal ramp test, with a 2-min warm-up and then increments of 30 W.min^−1^ in normoxia and 15 W.min^−1^ in hypoxia	A greater drop in HRmax in EIH athletes than in non-EIH athletes, despite the lack of a difference in SaO_2_ during hypoxic exercise
Chapman et al. (1999)	Eight EIH and six non-EIH endurance-trained athletes	Two maximal ramp tests in normoxia and hypoxia (FiO_2_ = 18.7%)	A running maximal ramp test, with a 2-min warm-up and then a 2% increment in the treadmill's slope every 2 min. The speed remained constant and was selected by the participant.	A greater drop in VO_2_max between normoxia and hypoxia in EIH athletes than in non-EIH athletes (despite a similar ΔSaO_2_) during hypoxic exercise. Whatever the condition, SaO_2_ was lower in EIH athletes.
Gaston et al. (2016)	Seven EIH and eight non-EIH athletes (runners or cyclists)	Two maximal ramp tests in normoxia and hypoxia (2150 m)	A cycling maximal ramp test, with a 3-min warm-up and then a 30 W.min^−1^ increment.	A greater drop in VO_2_max and HRmax in EIH athletes than in non-EIH athletes, despite the lack of a difference in SaO_2_ during hypoxic exercise.
Grataloup et al. (2007)	10 EIH and nine non-EIH cyclists	Two maximal ramp tests in normoxia and hypoxia (FiO_2_ = 15.1%)	A cycling maximal ramp test, with a 2-min warm-up and then a 0.33 W.min^−1^.kg^−1^ increment.	Greater drops in VO_2_max, HR_max_, and SaO_2_ in EIH athletes than in non-EIH athletes during hypoxic exercise
Raberin et al. (2019a)	15 EIH and 10 non-EIH athletes (runners and triathletes)	Two maximal ramp tests in normoxia and hypoxia (FiO_2_ = 15.3%)	A running maximal ramp test, with a 3-min warm-up and then a 1 km.h^−1^. min^−1^ increment.	A greater drop in VO_2_max, HRmax, and SaO_2_ in EIH athletes than in non-EIH athletes during hypoxic exercise
Verges et al. (2005)	11 EIH and nine non-EIH athletes (triathletes and ski touring)	Two submaximal exercise in normoxia and hypoxia (FiO_2_ = 15%)	Cycling for 10 min at 40% maximum power and (after a 2-min rest) 10 min at 60% maximum power.	No differences between EIH and non-EIH athletes in VO_2_, PaO_2_ or HR during submaximal exercise.

In another way, since the Mexico Olympics, scientists have been particularly interested in altitude training. The traditional “live high, train high” paradigm, i.e., a training camp situated at moderate altitude (1800–2500 m) is usually implemented two or three times a year (Millet et al., 2010). Historically, it was expected that the altitude-induced hematological response would increase O_2_ transport and thus aerobic performance back at sea level. However, the nature of other responses requires further investigation. The “live high, train low” (LHTL) altitude training mode was developed in which athletes live at moderate altitude but train at sea level or at low altitude (Levine and Stray-Gundersen, 1997). So, LHTL enables athletes to benefit from both the effects of chronic exposure to altitude and from maintaining a level of training intensity close to that used typically at sea level (Levine and Stray-Gundersen, 1997). In this initial study, Levine and Stray-Gundersen (1997) described significant hematological adaptations (notably elevated erythropoietin, hemoglobin, and hematocrit) associated with a gain in performance. Several follow-up studies gave essentially similar results (Hahn et al., 2001; Stray-Gundersen et al., 2001; Dehnert et al., 2002; Chapman et al., 2014) although other researchers observed no (or only small) hematological changes after LHTL (Ashenden et al., 1999, 2000). It is well-known that endurance performance (i.e., VO_2_max) does not depend solely on hematological parameters. Indeed, peripheral factors like exercise economy and/or buffering capacity (Gore et al., 2001, 2007; Saunders et al., 2004) might also contribute to post-LHTL performance. The initial LHTL model has evolved, with the development of new devices for artificially creating altitude conditions (i.e., normobaric hypoxia). At present, athletes no longer need to go up into the mountains to find a hypoxic environment. Regardless of the simulation method, not all endurance athletes respond in the same way to altitude training, and considerable inter-individual variations in responses have been documented (Chapman, 2013; Nummela et al., 2021). In this context, several methodological, training-related, and physiological factors have been identified but the putative impact of EIH on individual responses has never been investigated. Only a study evaluated the impact of intermittent hypoxia for 10 days (90 min^.^day^−1^, with FiO_2_ giving a SaO_2_ of 80%) on EIH athletes while others followed the same protocol under normoxic conditions (Marshall et al., 2008). The researchers found that exposure to intermittent hypoxia reduced the severity of EIH (94% after exposure, compared with 91% before). The increase in SaO_2_ might have been mediated by increased chemosensitivity at altitude, which in turn might increase hyperventilation (Katayama et al., 2001; Ainslie et al., 2003). Indeed, EIH athletes are thought to be poorly chemosensitive (Harms and Stager, 1995; Constantini et al., 2017; Granger et al., 2020). However, no improvements in VO_2_max or performance were reported. Thus, the interactions between EIH and altitude during the acclimatization phase remain to be explored.

## New Results and Potential Interaction Between EIH and Chronic Exposure to Altitude

There are few studies on this topic. It was recently reported that EIH athletes presented lower resting and maximal SpO_2_ values after 5 days of exposure to 2400 m (Durand et al., 2019). During this prolonged hypoxia, EIH athletes maintained a greater maximal cardiac output (vs. NEIH athletes), suggesting specific cardiovascular adaptations that enabled the achievement of the same VO_2_max. During the same protocol, EIH athletes exhibit an exacerbated oxidative stress at sea level compared to NEIH athletes while there was no between group difference after 1 day of exposure (Raberin et al., 2021). This result suggests that the impact of EIH on oxidative stress was blunted by acute altitude. However after 5 days of exposure, the reappearance of the between group difference suggest that EIH athletes may elicit a greater ventilatory acclimatization due to higher oxidative stress, since oxidative stress has been shown to modulate the hypoxic ventilatory response (Pialoux et al., 2009). However, the study failed to conclude on a role of oxidative stress at the onset of EIH due to its impact on pulmonary endothelial permeability (Nielsen, 2003).

**Figure 1:**
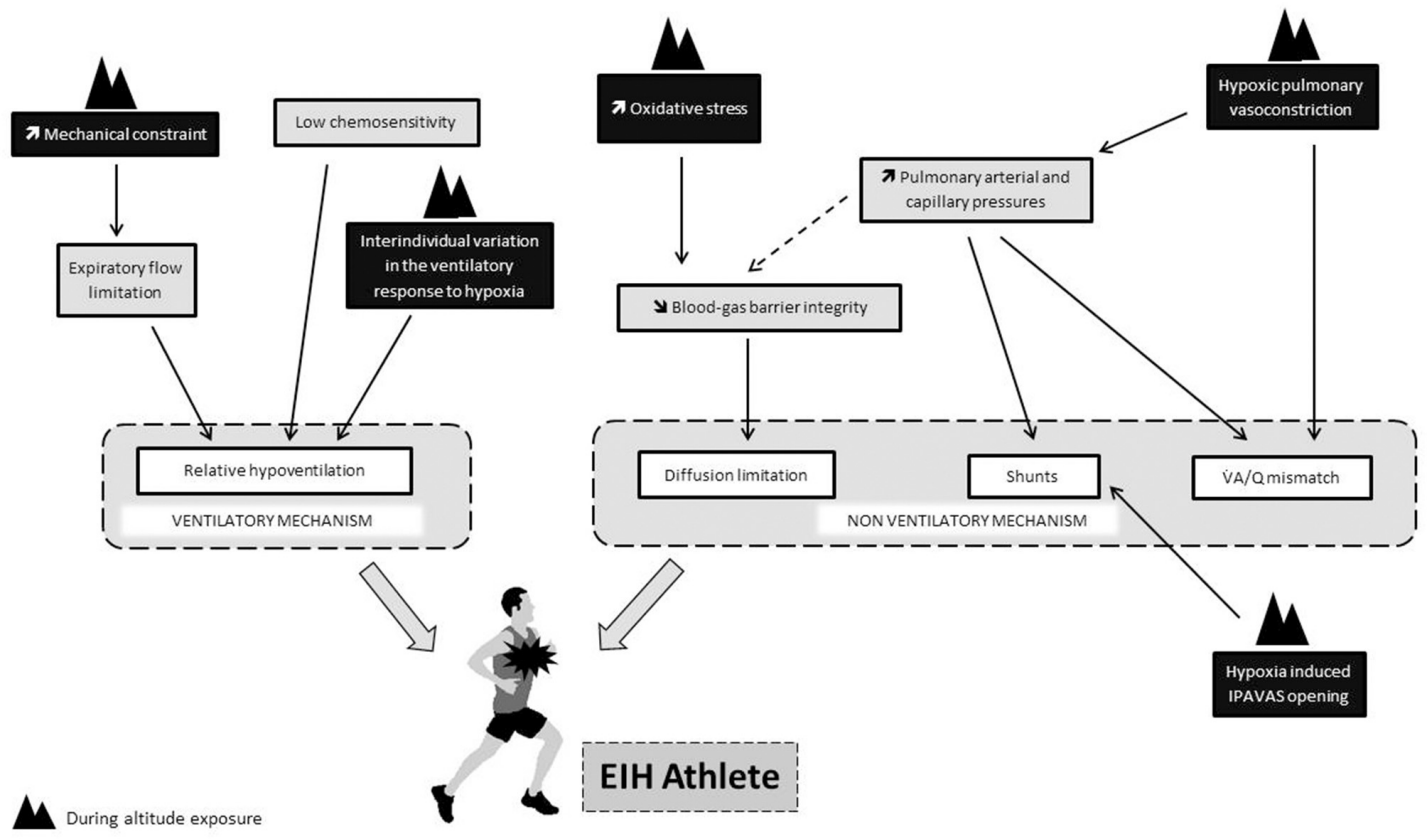
Putative impact of hypoxia (black rectangle) on physiological determinants thought to be involved in the onset of EIH at sea level (gray rectangle). Dotted line refers to the “stress failure theory.”.

One could also suspect that the mechanisms at the origin of EIH at sea level could be exacerbated during prolonged altitude exposure. The low chemosensitivity of EIH athletes could impact the kinetic of ventilatory acclimatation and be at the onset of a greater relative hypoventilation during exercise in hypoxia. An exacerbation of the ventilation/perfusion mismatch and extravascular pulmonary fluid movements may also occur during prolonged exposure. Indeed, pulmonary vessels constrict in response to alveolar hypoxia, this phenomenon known as hypoxic pulmonary vasoconstriction (HPV) aims to divert blood to better-oxygenated lung segments, thereby optimizing ventilation/perfusion matching and systemic O_2_ delivery (Swenson, 2013). HPV is known to persist during prolonged exposure to hypoxia and to increase pulmonary arterial pressure and pulmonary arterial resistance (Raberin et al., 2019b). These hemodynamic changes might be triggered intrapulmonary arteriovenous anastomosis (IPAVAS) recruitment leading to an arterial O_2_ desaturation due to shunt. Some evidences show that hypoxia could have a role in the opening of IPAVAS but whether O_2_ tension regulates IPAVAS remains unclear (Laurie et al., 2010; Lovering et al., 2015). Further studies are needed to understand how EIH changes during chronic exposure to altitude. Finally, the Figure 1 provides a summary of the physiological determinants of EIH in relation to the modalities of exposure to hypoxia.

## EIH: From Physiological Consideration to Training Monitoring

The roles of EIH following diffusion limitation (Chapman et al., 1999), excessive work of breathing (Amann et al., 2007), and increased pulmonary vascular resistance and arterial pressure (Eldridge et al., 2006; Faoro et al., 2009) are clear and raise the question of whether EIH has an additional role in the context of hypoxic training.

However, it seems essential to better understand EIH in ecological condition since athletes rarely reach maximal aerobic capacities during training. In addition, the course of EIH during altitude/hypoxic training and its consequences on training goals should be investigated. We can suggest different avenues to develop this knowledge: (i) monitor SpO_2_ during ecological training session to understand the frequency, duration, and severity of the hypoxemic episode and which kind of training might induce significant hypoxemia, (ii) evaluate whether EIH could impair or facilitate acclimatization to altitude and thereafter, (iii) investigate the putative role of EIH in the great variability of the response to altitude/hypoxia training whatever the method. In order to collect and analyze the large amounts of data and their interaction, openness to the science complex system is essential. In recent years, mathematics has been increasingly used in physiology (Lloyd et al., 2016; Pereira et al., 2018). The integration of sports science analysis with complex networks is called “complex sports analytics” and makes it possible to approach complex network structures as a very promising model prediction tool.

Individualization of the altitude dose is well-known to be the key of altitude/hypoxic training to ultimately reduce inter-individual variability. In the context, monitor the EIH during training has to be considered as a main factor of this individualization to find the best practice to increase performance.

## Author Contributions

The original conception of the work was conducted and the manuscript was drafted by FD and AR. Both authors read and approved the final manuscript.

## Conflict of Interest

The authors declare that the research was conducted in the absence of any commercial or financial relationships that could be construed as a potential conflict of interest.
